# Low-Cost Inorganic Strontium Ferrite a Novel Hole Transporting Material for Efficient Perovskite Solar Cells

**DOI:** 10.3390/nano12050826

**Published:** 2022-03-01

**Authors:** Ankush Kumar Tangra, Mohammed Benali Kanoun, Souraya Goumri-Said, Ahmed-Ali Kanoun, Kevin Musselman, Jaspinder Kaur, Gurmeet Singh Lotey

**Affiliations:** 1 Department of Physics, Sant Baba Bhag Singh University, Jalandhar 144030, India; aktangra@gmail.com; 2 Department of Physics, College of Science, King Faisal University, Al-Ahsa 31982, Saudi Arabia; mkanoun@kfu.edu.sa; 3 Department of Physics, College of Science, Alfaisal University, Riyadh 11533, Saudi Arabia; 4 Satellite Development Center, POS 50 Ilot T12, Bir-El Djir, Oran 31130, Algeria; aakanoun@cds.asal.dz; 5 Department of Mechanical and Mechatronics Engineering, University of Waterloo, Waterloo, ON N2L 3G1, Canada; kevin.musselman@uwaterloo.ca; 6 Dayanand Ayurvedic College, Jalandhar 144008, India; drjassisaggu@gmail.com; 7 Nano Research Lab, Department of Physics, DAV University, Jalandhar 144012, India; gsloteyz@gmail.com

**Keywords:** perovskite solar cells, strontium ferrite, transporting layers, band alignment

## Abstract

Perovskite solar cells attract significant interest due to their high-power conversion efficiencies. The replacement of charge-transporting layers using inorganic materials is an effective approach for improving stability and performance, as these materials are low-cost, highly durable, and environmentally friendly. This work focuses on the inorganic hole and electron transport layers (HTL and ETL), strontium ferrite (SrFe_2_O_4_), and zinc oxide (ZnO), respectively, to enhance the efficiency of perovskite solar cells. Favorable band alignment and high charge-collection capability make these materials promising. Experimental and computational studies revealed that the power conversion efficiency of the fabricated device is 7.80% and 8.83%, respectively. Investigating electronic properties and interface charge transfer through density functional theory calculations further corroborated that SrFe_2_O_4_ is a good HTL candidate. Our numerical device modeling reveals the importance of optimizing the thickness (100 nm and 300 nm) of the HTL and perovskite layers and defect density (10^16^ cm^−3^) of the absorber to achieve better solar cell performance.

## 1. Introduction

Remarkable progress was achieved in the field of perovskite solar cells, and Taylor et al., reported a power conversion efficiency of ~25.5% in 2021 [1]. P-type organic materials viz., poly(3-hexylthiophene-2,5-diyl) (P3HT); 2,2′,7,7′-tetrakis(N,N-pdimethoxyphenylamino)-9,9′-spirobifluorene (spiro-OMeTAD); poly[bis(4-phenyl)(2,4,6-trimethylphenyl)amine] (PTAA); and poly(3,4-thylenedioxythiophene) polystyrene sulphonate (PEDOT:PSS) were used as hole-transport layers (HTLs). However, organic HTL spiro-OMeTAD and PTAA have several shortcomings, including high production costs, possible thermal instability, low mobility, low conductivity, and poor stability in their pristine form [2,3], which limit their potential application in the commercialization and indispensable doping techniques involved in conductivity as well mobility, but increase the cost of production. Great efforts were made to develop novel inorganic HTLs with higher stability concerning low fabrication and material cost, high moisture, temperature, time, and exposure to sunlight and the ambient environment. The HTL can play a crucial role in shielding the perovskite layer from direct exposure to environmental effects and extracting holes from the perovskite absorber. Inorganic transporting layers have attracted significant attention for improving the efficiency and stability of these devices. There are several reports of using inorganic materials as the HTL to enhance both the stability and performance of perovskite solar cells. The use of NiO_x_ as the HTL and ZnO as the electron transport layer ETL resulted in high efficiency of 16.1% and stability [4]. You et al. and Liu et al. employed NiO_x_ as the HTL and achieved 14.60% and 14.11% power conversion efficiencies due to large carrier mobility, with the additional advantages of cheap production and high durability [5]. Other reported inorganic HTLs include CuI, KFeO_2_, CuScN, and Cu_2_O [5,6].

The ferrites of alkali metal may have high carrier mobility and stability compared to the aforementioned organic hole and electron transport materials. Ansari et al. reported 15.42% power conversion efficiency using Fe_3_O_4_ as a transporting layer [6]. Ferrites have distinctive physiochemical and optoelectronic properties and several superior assets, such as a tunable bandgap that can be easily tailored and is dependent on the structure and thickness of the transporting layer. In this regard, we are searching for a novel inorganic cost-effective, efficient, stable, dopant-free hole transport layer to achieve efficient perovskite solar cells. SrFe_2_O_4_ is a p-type semiconductor that meets this demand as SrFe_2_O_4_ is low cost, easily available, has high conductivity and mobility compared to the spiro-OMeTAD, PTAA, and many organic hole transport layers as well as energy band alignment with ETL and perovskite layers. Additionally, the inorganic nature of SrFe_2_O_4_ will reduce the energy losses at the interface and passivate the unavoidable defects present in the CH_3_NH_3_PbI_3_. It was previously investigated that SrFe_2_O_4_ cannot be oxygenated [7].

In this paper, strontium ferrite is introduced as a promising HTL candidate to enhance the efficiency of perovskite solar cells. Cost-effective spin-coating was adopted for the fabrication of the HTL, and experimental and theoretical studies were performed on devices with this new inorganic HTL. The influence of the SrFe_2_O_4_ HTL material on the photo-electrochemical properties of the device and operating mechanisms is also discussed.

## 2. Experimental

### 2.1. Material and Methods

#### 2.1.1. Materials

Research grade materials were purchased from the Loba-Chemie laboratory, Maharashtra, INDIA and used without purification. Ethylene glycol (C_2_H_6_O_2_), strontium nitrate (Sr(NO_3_)_2_), ferric nitrate (Fe(NO_3_)_3_⋅9H_2_O), citric acid monohydrate (C_6_H_8_O_7_), zinc acetate dihydrate (Zn(CH_3_CO_2_)_2_⋅2H_2_O), sodium hydroxide (NaOH), acetone (CH_3_COCH_3_), deionized water, isopropanol (CH_3_)_2_CHOH), and FTO glass were used as precursor materials. PbI_2_ and CH_3_NH_3_I were purchased directly from Merch for the fabrication of CH_3_NH_3_PbI_3_.

#### 2.1.2. Preparation of Sol of SrFe_2_O_4_

Figure S1a describe the schematics of the various steps involved in the fabrication of SrFe_2_O_4_ sol. In a typical synthesis process, 60 mL solvent, i.e., ethylene glycol, is divided into two parts (30 mL each). Firstly, ferric nitrate of 2 M (12.12 g) is dissolved in the first half of the ethylene glycol (30 mL) under continuous magnetic stirring at 30 °C temperature. On the other, strontium nitrate of 2 M (24.24 g) is dissolved in another half part of ethylene glycol (30 mL) at 30 °C. These two precursor solutions are allowed to stir (~30 min) until they become a clear solution. Afterward, the above solutions are mixed, and subsequently, citric acid 0.2 M (2.52 g) is added to it. The above mixture is allowed to continue stirring for 2 h at 50 °C. The brownish color sol of SrFe_2_O_4_ is formed, which will be further used for the fabrication of HTL.

#### 2.1.3. Preparation of ZnO Sol

Zinc acetate dihydrate, sodium hydroxide, and ethylene glycol are used as starting materials for the preparation of ZnO sol. Zn(CH_3_CO_2_)_2_·2H_2_O of 2 M (13.17 g) is dissolved in 30 mL of ethylene glycol under continuous stirring at 30 °C until it is completely dissolved. On the other hand, the second solution of NaOH having 2 M (2.4 g) concentration is prepared in ethylene glycol. This solution is added drop-wise in zinc acetate solution and stirred vigorously for 3 h at 30 °C. The formation of transparent and viscous sol indicates the formation of ZnO sol, and this will be further used for the deposition of the electron transport layer (ETL). The detailed description for the fabrication of ZnO sol is described in Figure S1b.

#### 2.1.4. Device Fabrications

**Figure 1:**
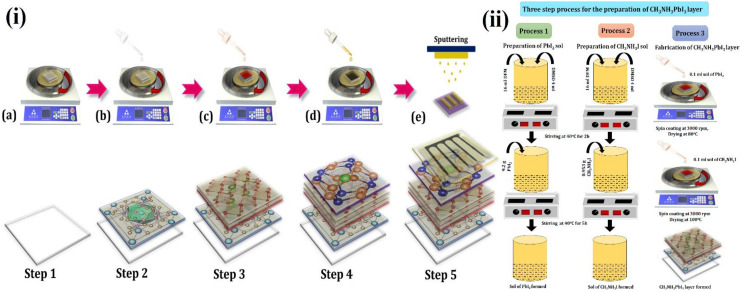
(**i**) Schematic of the fabrication process for perovskite solar cell. (**a**) Step 1: FTO Substrate is cleaned with different solvents. (**b**) Step 2: SrFe_2_O_4_ is spin-coated in ambient air. The fabrication process is shifted to the glove box. (**c**) Step 3: the CH_3_NH_3_PbI_3_ is coated onto the SrFe_2_O_4_ layer. (**d**) Step 4: ZnO is spin-coated over the perovskite absorber layer. (**e**) Step 5: Pt electrode is sputtered on the top of the device. (**ii**) Schematics of a three-step process for the fabrication of the CH_3_NH_3_PbI_3_ layer. Process 1 involved the preparation of PbI_2_ sol. Process 2 involved the preparation of CH_3_NH_3_I sol. Process 3 involved the formation of the CH_3_NH_3_PbI_3_ layer by spin coating technique onto FTO.

The detailed process for device manufacturing is elaborated in Figure 1. Pre-patterned FTO was purchased from Ossila, having substrates of 20 mm × 15 mm, thickness 1.1 mm, in which six small pixels with an area of 2.56 mm^2^ (1.6 mm × 1.6 mm) were patterned using electron beam deposition. The FTO was ultrasonically cleaned first with acetone, then isopropanol, and subsequently with ethanol, followed by UV ozone cleaning to remove impurities. A sol-gel method was used for the fabrication of the device (see Supplementary Materials, Figure S1).

**Figure 2:**
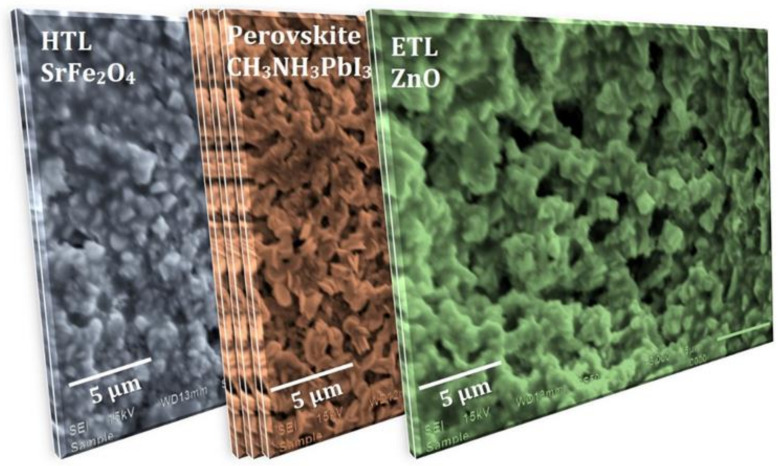
Top-view SEM images of the HTL (SrFe_2_O_4_), perovskite (CH_3_NH_3_PbI_3_), ETL (ZnO).

The volume of the sols used for the spin coating of each transporting layer (be it ETL, HTL, and perovskite) is 0.1 mL. These sols were filled in the micropipettes, and then 0.1 mL of it was poured on FTO each time for the deposition of ETL, the perovskite layer, and HTL (details given below).

i. Step 1: The first step for the fabrication of a solar cell device is to place the FTO on the substrate holder of the spin-coater. A total of 0.1 mL SrFe_2_O_4_ sol (40 mg/mL) was poured onto the FTO, and spin coating at 5000 rpm was conducted for 60 s. Then, the substrate was annealed at 100 °C for 20 min. This SrFe_2_O_4_ layer acts as a HTL.
ii. Step 2: A three-step process, Figure 1ii, was adopted for the coating of the perovskite layer (CH_3_NH_3_PbI_3_) [8]. Process 1 involved the preparation of PbI_2_ sol. For the preparation of PbI_2_ sol, 16 mL of N,N-Dimethylformamide (DMF) and 4 mL of dimethyl sulfoxide (DMSO) were mixed and allowed to stir for 2 h at 40 °C. Afterward, 9.2 g of PbI_2_ was added to this mixture, and this was allowed to stir for 5 h at 40 °C. This led to the formation of PbI_2_ sol. Process 2 involved the preparation of CH_3_NH_3_I sol. For this, again, 16 mL of DMF and 4 mL of DMSO were mixed and allowed to stir for 2 h at 40 °C. Subsequently, 0.953 g CH_3_NH_3_I was added, and this was allowed to stir for 5 h at 40 °C. The formation of CH_3_NH_3_I sol took place.
iii. Step 3: As explained in Figure 1i (Step 3) and Figure 1ii, (Process 3) involved the deposition of the CH_3_NH_3_PbI_3_ layer by spin coating technique onto SrFe_2_O_4_ coated FTO. The 0.1 mL of PbI_2_ sol (450 mg/mL) was poured onto SrFe_2_O_4_ and spin-coated at 3000 rpm. This was dried at 80 °C for 15 min, then 0.1 mL sol of CH_3_NH_3_I (50 mg/mL) was poured onto it, and spin-coating was carried out at 3000 rpm. Afterward, it was dried at 100 °C for 60 min. This would led to the formation of a perovskite layer (CH_3_NH_3_PbI_3_) on the surface of FTO. CH_3_NH_3_PbI_3_ is used as a perovskite layer because it is an ideal and superlative perovskite material since it absorbs light in the visible light regime; has a high dielectric constant, high carrier diffusion, and high Bohr radius; dissociates free charge carriers easily; allows tunable band engineering; has a low exciton binding energy ~2 meV and long carrier lifetimes [2].
iv. Step 4: The 0.1 mL ZnO sol (40 mg/mL) was poured on the above-fabricated substrate for the deposition of the ZnO layer that will act as ETL. The spin-coating was conducted at 3000 rpm and was dried for 10 min at 200 °C.
v. Step 5: The deposition of the Pt electrode was carried out onto the above-fabricated substrate using a sputtering process. The surface morphology of each layer of the device is presented in Figure 2, showing the large grain size and presence of voids.

### 2.2. Instrumentation

A PANalytical X’Pert PRO MRD X-ray diffractometer with λ = 1.54060 Å was utilized for structural analysis of the SrFe_2_O_4_, CH_3_NH_3_PbI_3,_ and ZnO. Rietveld refinement of X-ray diffraction (XRD) data was conducted by MAUD software for the identification of phase and lattice parameters. The optical properties (transmittance, bandgap) of the synthesized SrFe_2_O_4_ were measured using a UV-1800 Shimadzu UV-Visible spectrometer. Ultraviolet photoelectron spectroscopy (UPS) measurements were conducted using a Nexsa X-ray photoelectron spectrometer (XPS) system with a gas discharge lamp filled with He with the energy of 21.2 eV to calculate the electronic work function, valence band density, lowest unoccupied molecular orbital (LUMO), and highest occupied molecular orbital (HOMO) levels of the synthesized materials.

The current density versus voltage characteristics of the devices was recorded using a Keithley 2400 source meter under simulated AM 1.5 G solar irradiation (Newport, AAA solar simulator, 96,000, Xe lamp) at an intensity of 100 mW/cm^2^. The area of the fabricated devices was 0.0256 cm^2^. There were six pixels on each FTO substrate, and the devices were illuminated from the FTO side. The measurement of incident photon-to-current conversion efficiency (IPCE) was carried out using a computer-controlled IPCE system from Newport comprised of a Keithley 2400 source meter, monochromator, and Xe lamp. Time-resolved photoluminescence spectra (TRPL) of the devices were measured using an Edinburgh Instruments FLS920 at an excitation wavelength of 330 nm. The impedance spectra of the fabricated solar cell were measured using Autolab at a constant forward applied bias of 0.2 and 0.8 V and frequency between 400 kHz and 0.05 Hz.

### 2.3. First Principle Calculations

Density functional theory (DFT) calculations were carried out using the Quantum Atomistix ToolKit (QuantumATK) package [9]. The local combination of the atomic orbitals approach and the Perdew, Burke, Ernzerhof (PBE) generalized gradient approximation (GGA) as the exchange-correlation functional were adopted [10]. Norm-conserving PseudoDojo pseudopotential with a medium basis set and a mesh cut-off energy of 105 Ha was employed [11]. The Brillouin region was sampled using a 4 × 5 × 3 and 4 × 5 × 7 Monkhorst-Pack’s special k-point grid during geometry optimization and electronic property calculation. The self-consistent calculation converged and was completed with a minimum of 10^−6^ Ha difference in the total energy. The interatomic forces are relaxed to 0.05 eV/Å using the limited-memory Broyden–Fletcher–Goldfarb–Shanno (LBFGS) algorithm. The spin-orbital coupling (SOC) contribution was considered during the calculation of the electronic properties owing to the strong relativistic effect caused by the Pb element. GGA + U calculations were conducted using U = 4 eV for the Fe atom to consider the strong correlations [12].

### 2.4. Device Simulation

Numerical simulations were carried out using the Solar Cell Capacitance Simulator for one-dimensional solar cell (SCAPS-1D) software under the AM 1.5 solar spectrum at a 100 mW/cm^2^ light intensity to compute current density versus applied voltage (J-V) characteristics at 300 K [13]. The perovskite solar cell was considered a thin film device comprised of a stack of layers consisting of ETL (ZnO), absorber (perovskite), and HTL (SrFe_2_O_4_) and was defined by thickness, doping, and other physical parameters in SCAPS-1D. The software utilizes the Poisson’s and carrier continuity equations under the boundary conditions for J-V characteristics. Details are given in the Supplementary Materials. Such methods were extensively used to explore the device properties of photovoltaics based on perovskites [14,15,16,17].

## 3. Results

**Figure 3:**
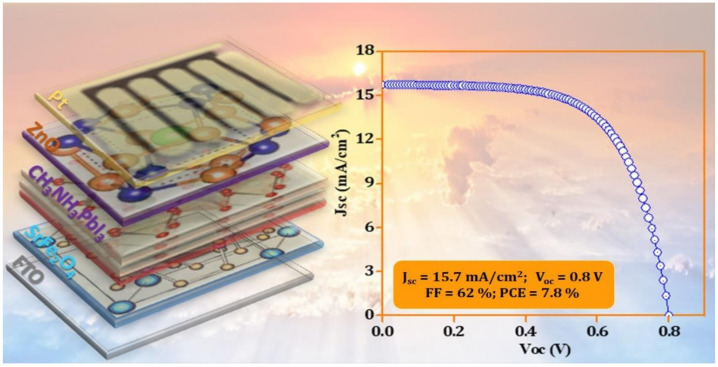
Schematic of fabricated devices representing the various layers FTO/SrFe_2_O_4_/CH_3_NH_3_PbI_3_/ZnO/Pt and current density versus applied voltage (J-V) characteristics curve of the fabricated device under 1.5 AM illumination.

**Figure 4:**
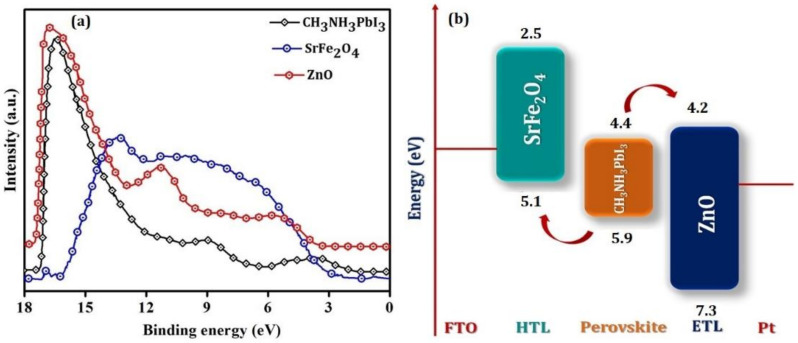
(**a**) Ultraviolet photoelectron spectra for SrFe_2_O_4_, CH_3_NH_3_PbI_3,_ and ZnO layers and (**b**) energy band alignment of these functional layers in the device.

The photo-conversion parameters for the best-performing device are shown in Figure 3 and were Jsc = 15.7 mA/cm^2^, V_OC_ = 0.80 V, and FF = 62%. The simulated parameters are presented in Table 1 and were 23.3 mA/cm^2^, 1.0 V, and 40%, respectively, which are in reasonable agreement with the experimental parameters. Moreover, a power conversion efficiency of 7.80% was obtained experimentally, which is close to the simulated value of 8.83%. This agreement exhibits evidence to extend the validity of the entire material parameters used in our simulation for defining the layers included. The energy bands of the perovskite, SrFe_2_O_4_, and ZnO layers in the device were determined by UPS (Figure 4a,b). The lowest unoccupied molecular orbital (LUMO) and highest occupied molecular orbital (HOMO) energy levels (conduction band/ valance band) of the CH_3_NH_3_PbI_3_ were found to be 4.4 eV, 5.9 eV.

The conduction band (CB) and valence band (VB) energy levels of the charge transporting layers, SrFe_2_O_4_ and ZnO, are 2.5 eV, 5.1 eV, and 4.2 eV, 7.3 eV, respectively. The VB of SrFe_2_O_4_ and CB of ZnO is well aligned with the VB and CB of the perovskite absorbing layer. The difference in the CB of CH_3_NH_3_PbI_3_ and ZnO is 0.2 eV, while the VB of CH_3_NH_3_PbI_3_ and SrFe_2_O_4_ is 0.8 eV. The band alignment of the transporting layers is very important for the injection of electrons and holes from the perovskite absorber layer as well as for the blocking of electrons and holes in the reverse direction [18,19].

The observed value of Voc may be attributed to the use of inorganic SrFe_2_O_4_ as HTL. The VB and CB positions of the SrFe_2_O_4_ are such that effective hole transfer from the perovskite and effective electron blocking is expected. The offset between the SrFe_2_O_4_ and perovskite VB (5.9–5.1 = 0.8 eV) is fairly large, which might explain the modest Voc of 0.8 V achieved.

The band engineering can be regarded as a future task for the improvement of the value of VB offset between the SrFe_2_O_4_ and perovskite. The increase in the value of the bandgap of SrFe_2_O_4_ from 2.6 eV may decrease the VB offset and thereby may increase the Voc that will further enhance the PCE parameters. The thickness of the HTL, ETL, and light-absorbing layer may also be beneficial play a very crucial role. There may be two reasons behind this, firstly, the adequate alignment of the bands of SrFe_2_O_4_ and perovskite materials [5]. Secondly, the valance band of HTL results in ohmic contact with the perovskite material that results in the high potential difference between the HTL and ETL [5]. The height of the pyramids is directly proportional to the root means square roughness of the substrate, Figure S3. The higher the height of the pyramids, as in our case, the higher the roughness. Hossain et al. (2018) reported that surface roughness leads to variations in the optical parameters and subsequently PCE [18].

The high surface roughness increases the interface resistance and de-accelerates the charge transportation. A high surface roughness was observed for the transporting layer. This may have led to a decrease in the efficiency because high surface roughness leads to a high recombination of electrons and holes that may subsequently lead to a decrease the efficiency. Figure S2 confirm the presence of a large number of pinholes in the ETL and light-absorbing layers compared to HTL. The observation of low V_oc_ is due to the high value of the root mean squares (RMS) parameters of the surface roughness of the ETL/HTL.

Furthermore, the interface between the ETL/Perovskite layer/HTL is inhomogeneous and anisotropic resulting in surface roughness, and the latter is the main source of optical and electrical effects. The observation of small grain size, Figure S2, can be seen in the ETL, HTL, and perovskite layers that increase the crystal grain boundaries, Figure S2, thereby increasing the photo-induced charge carrier recombination resulting in low FF [19,20,21,22,23]. The surface roughness observed is of the order of 10’s of µm resulting in the dispersion of the escape depth of the collected photoelectron and reducing the value of Voc and PCE. You et al. (2016) suggested that organic HTLs such as PEDOT: PSS have a high concentration of pinholes, non-uniform surface morphology, and poor interface connection that may degrade the power conversion parameters [5]. In contrast, the use of inorganic HTL overcomes all the above due to its superior properties compared with the organic one. The observed value of Jsc may be attributed to the strong hole and electron blocking nature of ZnO ETL and SrFe_2_O_4_ HTL from its deep VB and CB, respectively, along with the optical spacer effect [5].

**Figure 5:**
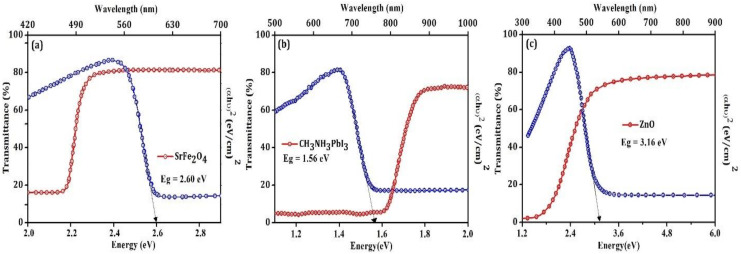
Transmittance spectra and Tauc’s relation for (**a**) SrFe_2_O_4_ having 2.60 eV bandgap. (**b**) CH_3_NH_3_PbI_3_ has 1.56 eV bandgap and (**c**) ZnO has 3.16 eV bandgap. The transmittance for all the layers of the device is above 70%.

Figure 5 reveal that the transmittance for the HTL, ETL, and perovskite materials is above 80%, 70%, and 70%, respectively. The high value of transmittance for HTL in the visible range of the spectrum allows the light to reach the perovskite materials without it being absorbed by HTL. It is necessary to reduce the series resistance and increase the shunt resistance to improve the performance of perovskite solar cells. The energy band gap of HTL may result in the observed open-circuit voltage, Figure 4, and may generate a large electrical field that leads to strong band bending between the perovskite absorber and HTL interface [21]. This electric field pushes the minority carrier in the perovskite layer away from the HTL interface and successfully decreases the recombination through the interfacial defects [21]. The main role of HTL and ETL is to extract and transport electrons and holes [22]. HTL serves as an electron blocking layer, whereas ETL is a hole blocking layer and conquers the charge recombination in the perovskite solar cell. Additionally, HTL aids in the enhancement of hole transfer efficiency; protecting the electrode contact interface from degradation; assisting in enhancing the open-circuit voltage; providing a physical barrier between conductive glass (p-i-n configuration) or between the electrode and perovskite layer direct influence on the photovoltaic parameter (V_oc_, FF and J_sc_) [24]. SrFe_2_O_4_ is a direct bandgap semiconductor, posessing an optical bandgap of 2.60 eV. Normally the materials used for the HTL have a bandgap above 3 eV [5], but the bandgap of the SrFe_2_O_4_ is less, and this may decrease the value of the Jsc and result in low power conversion efficiency. ZnO with a direct bandgap semiconductor of around 3.85 eV, electron diffusion coefficient (1.7 × 10^−4^ cm^2^·s^−1^), and electron mobility (205–300 cm^2^ V^−1^·s^−1^) would be the reason for better power conversion efficiency [25]. The transportation of the hole and electrons depend on the Fermi levels of the HTL, ETL, and perovskite materials. Additionally, the hole mobility of the HTL should be at least 10^−3^ cm^2^·V^−1^·s^−1^ along with high stability at room temperature. It is classically proposed that HTL should have high conductivity and high mobility that may result in the high-power conversion efficiency of the perovskite solar cell [24]. ZnO as ETL is deposited on the top of the device since it is transparent and has strong resistance to the surrounding environment, oxygen, and vapor in the air.

**Figure 6:**
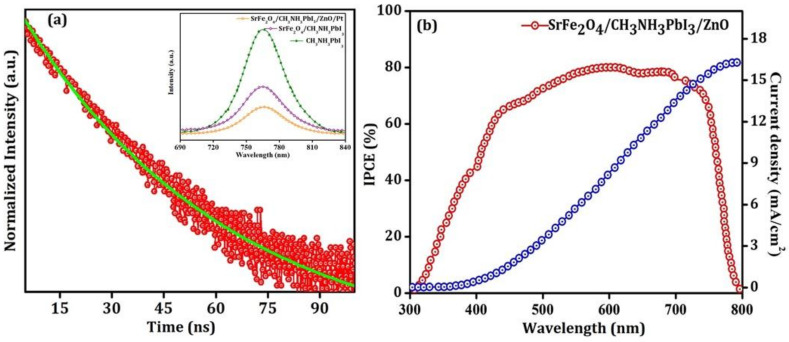
(**a**) Time-resolved photoluminescence spectra of the p-i-n planar fabricated device, the inset shows the energy-resolved PL and (**b**) Incident photon-to-current conversion efficiency (IPCE) spectra and integrated current density (Isc) of the device.

It is known that the properties of the HTL and ETL are intrinsically related to their HOMO and LUMO. Figure 4b confirm that the value of the HOMO and LUMO for ETL (ZnO) and HTL (SrFe_2_O_4_) are close enough to the conduction and valance band of the active perovskite absorber material; this allows the flow of the generated electrons and holes. Table S1 confirm that the values of the mobilities of the holes and electrons in HTL and ETL, respectively, are high to compensate for the loss that occurs throughout the charge transportation to the FTO and Pt electrodes. The inorganic nature of the HTL and ETL helps to reduce the impact of environmental damages such as humidity, oxygen, temperature, and illumination [26,27]. The inset of Figure 6 confirms the strong quenching of the photoluminescence intensity for CH_3_NH_3_PbI_3_, SrFe_2_O_4_/CH_3_NH_3_PbI_3_, and FTO/SrFe_2_O_4_/CH_3_NH_3_PbI_3_/ZnO/Pt. This quenching of the peak confirms the capability of SrFe_2_O_4_ to extract the charge carrier from the perovskite absorber towards the electrode. Charge recombination takes place at the interface of layers that may occur as a result of incomplete coverage of the perovskite layer. All the samples exhibit PL emission peaks at 765 nm arising from the perovskite and are consistent with the reported results. The interface between HTL/perovskite results in a significant quench in the PL spectra owing to the more effective extraction of holes and confirming the high quality of the film. This further establishes the intimate contact with the perovskite absorber layer and the high mobility of HTL. Charge dynamics were studied by time-resolved photoluminescence (TRPL) spectra, as shown in Figure 6a. The experimental data were fit with a bi-exponential function:

(1) $$f\left(t\right)\;=\sum_{i}A_{1}e^{t/\tau_{1}}\;+\;A_{2}\;e^{t/\tau_{2}}\;+\;K$$

where, τ_1_, τ_2_, *A*_1_, *A*_2_ are decay times and decay amplitudes, and K is a baseline offset constant. The measured decay times were τ_1_ = 20.9 ns and τ_2_ = 59.8 ns. Here, τ_1_ is ascribed to the electron and hole extraction across the interface layer, whereas τ_2_ is the radiative recombination rate of the charge carriers. This further affirms that energetic injection of holes into HTL and decrease in the rate of recombination and optimal charge transfer. The small value of the τ_1_ confirms that the fabricated device has efficient hole extraction capability, and it considerably decreases the trap-induced non-radiative carrier recombination and thereby reduces photocurrent loss [28]. The small number of grain boundaries observed in perovskite materials, as shown in Figure 2 and Supplementary Materials, reveal the less corrosive effect of grains towards photo-conversion efficiency. The observation of a short lifetime, 20.9 ns, results in less electron-hole recombination, and this leads to a high accumulation of holes in HTL and electrons in ETL.

The incident photon-to-current conversion efficiency (IPCE) spectrum of the device in Figure 6b show that the device produces photocurrent up to 770 nm. The IPCE spectrum is in good agreement with the transmittance spectra (Figure 5) of the materials. The IPCE is 79.90% at 585 nm, Figure 6b. The I_SC_ value under AM 1.5 G solar irradiation is 16.3 mA/cm^2^, which correlates with the J-V study. The observed value of the IPCE spectra is due to the crystal boundary passivation and reduction of the surface defects, Table S1 [29]. The high roughness of the absorbing perovskite layer in Figures S2 and S3 resulted in a decrease in overall IPCE over a visible spectrum; this dropdown corresponding to J_sc_ and decreasing the PCE.

Bulk cells of pseudo-cubic CH_3_NH_3_PbI_3_ and monoclinic SrFe_2_O_4_ were considered, and their lattice parameters and atomic positions were optimized. The obtained equilibrium parameters for CH_3_NH_3_PbI_3_ were 6.343 Å, which are in good agreement with the experimental value of a = 6.391 Å [30]. The calculated lattice parameters a = 7.9323 Å, b = 9.2339 Å, c = 10.7070 Å for SrFe_2_O_4_ are well reproducible with X-ray diffraction values, Figure S4. The electronic band structures of CH_3_NH_3_PbI_3_ and SrFe_2_O_4_ are determined and displayed in Figure S5. The band structure of CH_3_NH_3_PbI_3_ was determined with DFT-1/2 and SOC, revealing that it exhibits a direct energy bandgap at the high symmetry Γ- point of magnitude around 1.6 eV, which is more consistent with our experimental results as well as previous theoretical works [31].

**Figure 7:**
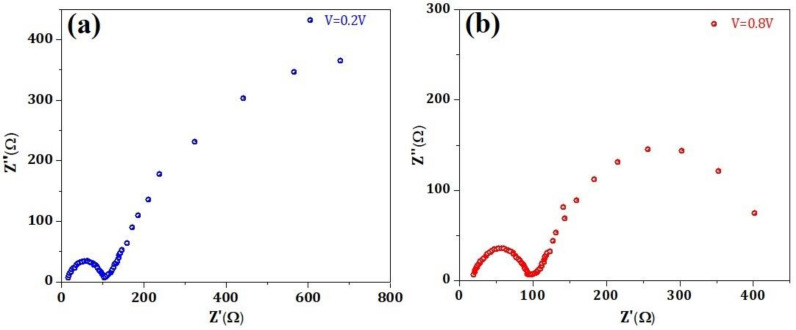
Impedance spectra for the fabricated solar cell at (**a**) 0.2 V and (**b**) 0.8 V applied bias.

Figure 7 show the characteristic impedance spectra of the device at applied voltages 0.2 V (Figure 7a) and 0.8 V (Figure 7b) during working environments under one sun illumination. In both cases, an arc-type curve was observed at high frequencies due to the transport in HTL (SrFe_2_O_4_). On the other hand, at low frequencies, a typical classical behavior was noticed. The impedance spectra for solar cell devices generally have three arcs at low, intermediate, and high frequencies corresponding to dielectric relaxation, charge recombination, and selective contact charge transfer [20,31,32,33,34]. However, Figure 7 show that only two arcs were observed for low frequency and high frequency, revealing the charge transportation and separation at the interface between the perovskite (CH_3_NH_3_PbI_3_) layer and HTL, respectively. The observed characteristics clearly indicate that the charge transportation is dominated by charge carriers that are further coupled with recombination across the active layer [20,31,32,33,34]. This revealed the ambipolar charge transportation in the solar cell device vis à vis coupled electron-hole transport, which is ambipolar diffusion. It is not possible to explicitly say or predict the nature and the type of the carrier from the impedance spectra as an electrical response is symmetrical for the electrons as well as holes [20,31,32,33,34].

**Figure 8:**
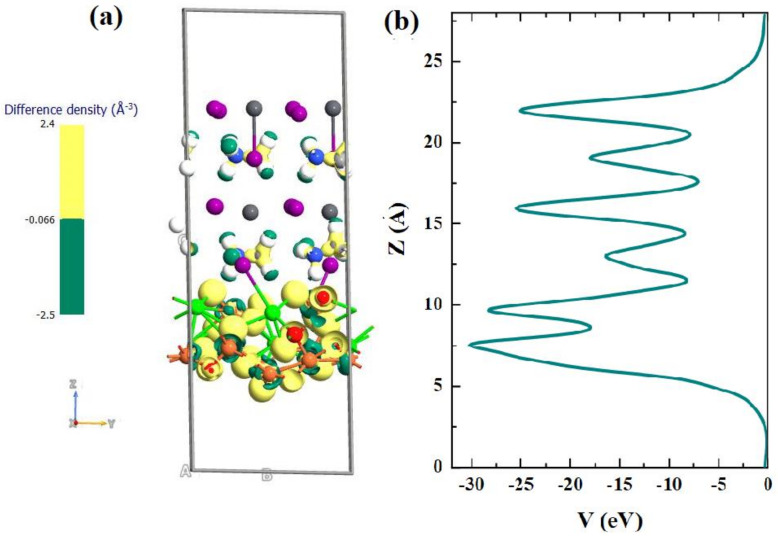
(**a**) Isosurface charge density difference, isovalue is set at 0.06 e/Å^3^ and (**b**) plane-averaged effective potential plots for the SrFe_2_O_4_/CH_3_NH_3_PbI_3_.

**Figure 9:**
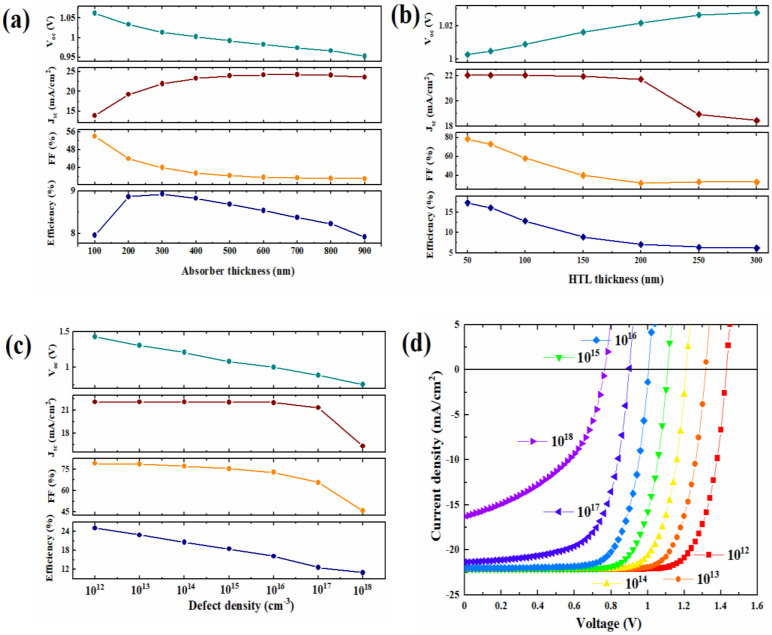
Effect of thickness layers on solar cell parameters of (**a**) absorber perovskite, (**b**) hole transport material, (**c**) device parameters, and (**d**) J-V curves with varying defects density in the perovskite absorber layer.

The spin-resolved band structure and the total partial density of states were calculated using GGA + U, as illustrated in Figure S5. The energy bandgap of the SrFe_2_O_4_ in the spin-up channel is well above 3.0 eV, which is more consistent with our obtained experimental data. To obtain insight into the interfacial transport mechanism of the fabricated device, the difference in charge density and effective potentials are addressed using SOC effect and DFT-1/2 levels of approximation, as shown in Figure 8. The geometry structure of the SrFe_2_O_4_(001)/CH_3_NH_3_PbI_3_ (001) interface is formed from the (001) surfaces in the slab models of SrFe_2_O_4_ and CH_3_NH_3_PbI_3_ with CH_3_NH_3_I-termination along with the c-axis. Interface models are built by expanding four layers of thick slabs of perovskite in a CH_3_NH_3_I-terminated surface and two layers slabs of SrFe_2_O_4_, Figure 9a. The interface adhesion energy is defined as the opposite of the energy requested to separate the surfaces that form the interface, which is obtained from the expression [32]:

(2) $$E_{adh}=E_{interface}-E_{CH_{3}NH_{3}PbI_{3\;surface}}-E_{SrFe_{2}O_{4\;surface}}$$

where $E_{interface}$, $E_{CH_{3}NH_{3}PbI_{3}\;surface}$, and $E_{SrFe_{2}O_{4\;surface}}$ represent the total energies of the interface, perovskites, and SrFe_2_O_4_ in the interface lattice, respectively. The interface adhesion energy for the SrFe_2_O_4_/CH_3_NH_3_PbI_3_ is found to be negative, i.e., −2.94 meV, demonstrating the investigated interface is stable and form easily.

To visualize the charge transfer, the charge density difference is computed between the interface and the surface model:

(3) $$\Delta \rho =\rho_{interface}-\rho_{CH_{3}NH_{3}PbI_{3\;surface}}-\rho_{SrFe_{2}O_{4\;surface}}$$

where $\rho_{interface}$, $\rho_{CH_{3}NH_{3}PbI_{3\;surface}}$, and $\rho_{SrFe_{2}O_{4\;surface}}$ denote the charge density of the interface, perovskite surface, and SrFe_2_O_4_, respectively. The yellow and green areas indicate a positive value of Δ*ρ* and a negative Δ*ρ* corresponding to the charge accumulation and depletion regions, respectively, Figure 9a.

The charge density difference plot of SrFe_2_O_4_/CH_3_NH_3_PbI_3_ interface shows strong charge accumulation represented by the yellow region is mainly on oxygen atoms of SrFe_2_O_4_ interface and on carbon of the CH_3_NH_3_I surface termination. It also shows the presence of a charge-deficit region on the H of the CH_3_NH_3_I surface termination and the excess charge electron depletion regions on the Fe atoms. These results are obtained by the charge redistribution at the interface. Moreover, the plane average effective potential of CH_3_NH_3_PbI_3_ layers shows a lower level than that of SrFe_2_O_4_ surface, as shown in Figure 9b. It can be possible to facilitate electron transfer and accumulation between the CH_3_NH_3_PbI_3_ and SrFe_2_O_4_ interface. The simulated solar cell configuration is the p-i-n architecture of FTO/SrFe_2_O_4_/perovskite/ZnO/Pt. The input parameters of materials used in numerical simulations are summarized in Table S1. Simulations were performed to optimize the absorber and HTL thicknesses. The perovskite thickness was changed from 100 nm to 1000 nm while keeping the HTL thickness constant at 250 nm. It can be seen from Figure 9a that the calculated J_sc_ rises with an increase in the absorber thickness to 24.0 mA/cm^2^ at 500 nm and saturates thereafter.

Moreover, V_oc_ and FF slightly drop as the thickness of the perovskite increases due to increased charge carrier recombination and series resistance in the thick absorber layer. Figure 9a illustrate that the simulated device efficiency rises and reaches the highest value of 9% at an absorber thickness of 300 nm, then decreases when the perovskite layer thickness is larger than 300 nm. Thus, the value of 300 nm is obtained as the optimal absorber thickness in this device architecture. The thickness of the SrFe_2_O_4_ HTL introduced in this study is optimized by varying it from 50 nm to 300 nm, while the thickness of the perovskite layer is kept constant at the optimized value of 300 nm, as illustrated in Figure 9b. It is observed in Figure 9b that an increase in the HTL thickness reduces the PCE from 17% to 6%. Similar behavior was observed for the FF. On the other hand, the V_oc_ is increased while the J_sc_ is slightly reduced to 18 mA/cm^2^ when the HTL thickness increases.

These results suggest that the thickness of the SrFe_2_O_4_ HTL should be minimized to enhance device performance. However, this must be balanced with the ability to produce a continuous, pinhole-free HTL, such that 50 nm was the smallest thickness considered here. In addition, the effect of the absorber defect density (N_t_) is also investigated. Figure 9c,d show the J-V curves and solar cell performance parameters for various values of defect density in the absorber perovskite layer. It is found from Figure 9c that V_oc_ and PCE monotonically dropped after increasing the density defect. Similar behavior was observed for J_sc_ and FF with a saturation value at 22.04 mA/cm^2^ and almost constant at 73% for density defects less than 1 × 10^16^ cm^−3^. It is observed that the J_sc_ and FF are rapidly reduced when the Nt increases from 10^17^ cm^−3^ to 10^19^ cm^−3^. This is owing to creating many recombination centers in interface and interior solar cells, which increases the carrier’s recombination rate. The calculated results correlate with the ones revealed in the literature [17]. Therefore, controlling the Nt under ~10^16^ cm^−3^ is a critical factor in achieving the high-efficiency CH_3_NH_3_PbI_3_ based perovskite solar cells. To obtain a deeper insight into the absorber layer’s defect density on solar cell performance, we analyzed the influence of defect density on the recombination rate using the Shockley–Read–Hall recombination model as given by Equation (S4).

**Figure 10:**
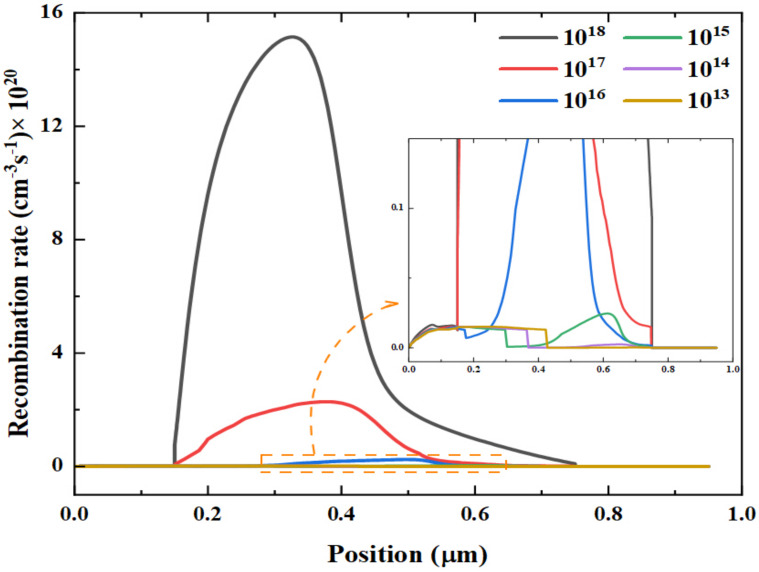
Effect of changing recombination rate with depth for different defect densities.

It can be observed from Figure 10 that the recombination rate increases with increasing the defect density, indicating the considerable destructive impact of the absorber defect level on the recombination rate. It can be seen that at a defect density value of 10^16^ cm^−3^, the carrier recombination is not apparent. In contrast, more carriers are captured by traps when the density of trap states reached 10^18^ cm^−3^, resulting in the raised recombination rate at the HTL and absorber interface. To define the range of the diffusion length needed at the absorber layer, the diffusion length of charge carriers is calculated using Equations (S7)–(S9) and represented as a function of defect density in the absorber layer (Figure S6). It is observed that the diffusion length is strongly affected by the changes in the defect density playing an important parameter to affect the efficiency of solar cell devices. It can be concluded that the estimated value of diffusion length for the charge carriers in the absorber layer is found to be 1.137 µm, which corresponds to the optimum value of the defect density for CH_3_NHPbI_3_ (10^16^ cm^−3^).

## 4. Conclusions

In summary, this study demonstrated the use of inorganic HTL and ETL in the inverted p-i-n configuration of perovskite solar cells with excellent photovoltaic performance. These transporting layers are low-cost and have high performance. Theoretical calculation suggests that the power conversion efficiency of the designed structured solar cell is 8.83%, while its experimental value is 7.80%. The value of the various power conversion parameters was found to be J_sc_ 15.7 mA/cm^2^ V_oc_ 0.80 V, confirming the low recombination of electrons and holes, thereby validating the effectiveness of the electrons and holes blocking layers in the perovskite solar cell. UPS and optical investigation confirmed that the value of the bandgap were 2.60 eV, 1.56 eV, and 3.16 eV for SrFe_2_O_4_, CH_3_NH_3_PbI_3_, ZnO, whereas the HOMO and LUMO energy bands were 2.46 eV, 5.0 eV; 4.34 eV, 5.91 eV; and 4.17 eV, 7.33 eV, respectively. The charge dynamics study confirmed that the decay time of the charge carrier for the fabricated solar cell was τ_1_ 20.9 ns and τ_2_ 59.8 ns, reflecting the rate of electrons and holes extraction and recombination across the interface layer of the perovskite solar cell. In addition, a comprehensive analysis was carried out using the first principle calculations and device simulation. Our DFT findings reveal that SrFe_2_O_4_ is considered an outstanding candidate for HTL. This is owing to the suitable bandgap, band alignments of HTL, ETL, and perovskite, and enhanced charge transfer. SCAPS simulation of the ideal device with inverted architecture exhibited the impact of the thickness of SrFe_2_O_4_ and perovskite on solar cell performance, proving that there was an improvement of photovoltaic performance by 12% under 100 nm and 300 nm, respectively. These results encourage a new approach and process using inorganic HTL and ETL toward the low-cost manufacturing of solar cells with better efficiency.

## Figures and Tables

**Table 1:** Properties of transporting layers and photo-conversion parameters of solar cell devices.

Materials	Structure	Optical Band Gap (eV)	UPS Spectra		Solar Cell Performance		
			LUMO	HOMO	Parameters	Experimental	Theoretical
SrFe_2_O_4_	Monoclinic	2.6	2.5	5.1	J_sc_ (mA/cm^2^)	15.7	23.3
CH_3_NH_3_PbI_3_	Cubic	1.6	4.4	5.9	V_oc_ (V)	0.8	1.0
ZnO	Cubic	3.2	4.2	7.3	FF (%)	62.0	40.0
-	-	-	-	-	PCE (%)	7.8	8.8

## Data Availability

Not applicable.

## Supplementary Materials

The following supporting information can be downloaded at: https://www.mdpi.com/article/10.3390/nano12050826/s1. References [35,36,37,38,39,40] are cited in the Supplementary Materials.
