# Supplementary material for: Low-Cost Inorganic Strontium Ferrite a Novel Hole Transporting Material for Efficient Perovskite Solar Cells
Source: Nanomaterials (Basel). 2022 Mar 1;12(5):826. doi: 10.3390/nano12050826 (PMC8912847; doi:10.3390/nano12050826)
Supplement: Supplementary file 1 [file nanomaterials-12-00826-s001.zip › nanomaterials-1585483-supplementary.pdf]

Supplementary Information

Low-Cost Inorganic Strontium Ferrite a Novel Hole Transporting Material for Efficient Perovskite Solar Cells

Ankush Kumar Tangra [1], Mohammed Benali Kanoun [2], Souraya Goumri-Said [3,*], Ahmed-Ali Kanoun [4], Kevin Musselman [5], Jaspinder Kaur [6] and Gurmeet Singh Lotey [7]

1 Department of Physics, Sant Baba Bhag Singh University, Jalandhar 144030, India; aktangra@gmail.com
2 Department of Physics, College of Science, King Faisal University, Al-Ahsa 31982, Saudi Arabia;
mkanoun@kfu.edu.sa
3 Department of Physics, College of Science, Alfaisal University, P.O. Box 50927, Riyadh 11533, Saudi Arabia; sosaid@alfaisal.edu.
4 Satellite Development Center, POS 50 Ilot T12, Bir-El Djir, Oran, Algeria, aakanoun@cds.asal.dz.
5 Department of Mechanical and Mechatronics Engineering, University of Waterloo, Waterloo, ON, Canada N2L 3G1; kevin.musselman@uwaterloo.ca.
6 Dayanand Ayurvedic College, Jalandhar 144008, India; drjassisaggu@gmail.com
7 Nano Research Lab, Department of Physics, DAV University, Jalandhar 144012, India; gsloteyz@gmail.com
* Correspondence: sosaid@alfaisal.edu; Tel.: +966-11-215-8984

2.1.1 Materials

Research grade materials have been purchased from the Loba-Chemie laboratory and used without purification. Ethylene glycol ($C_2H_6O_2$), strontium nitrate ($Sr(NO_3)_2$), ferric nitrate ($Fe(NO_3)_3.9H_2O$), citric acid monohydrate ($C_6H_8O_7$), zinc acetate dihydrate ($Zn(CH_3CO_2)_2.2H_2O$), sodium hydroxide (NaOH), acetone (CH_3COCH_3), deionized water, isopropanol $(CH_3)_2CHOH$), FTO glass are used as precursors materials. PbI_2 and CH_3NH_3I have been directly purchased from the Merch for the fabrication of $CH_3NH_3PbI_3$.

2.1.2 Preparation of sol of $SrFe_2O_4$

Figure S1 (a) describes the schematic of the various steps involved in the fabrication of $SrFe_2O_4$ sol. In a typical synthesis process, 60 mL solvent, i.e., ethylene glycol is divided into two parts (30mL each). Firstly, ferric nitrate of 2M (12.12 g) is dissolved in the first half of the ethylene glycol (30mL) under continuous magnetic stirring at 30°C temperature. On the other strontium nitrate of 2M (24.24g) is dissolved in another half part of ethylene glycol (30mL) at 30°C. These two

precursor solutions are allowed to stir (~30 minutes) till these became a clear solution. Afterward, the above solutions are mixed and subsequently, citric acid 0.2 M (2.52g) is added to it. The above mixture is allowed to continue stirring for 2h at 50°C. The brownish color sol of $SrFe_2O_4$ is formed which will be further used for the fabrication of HTL.

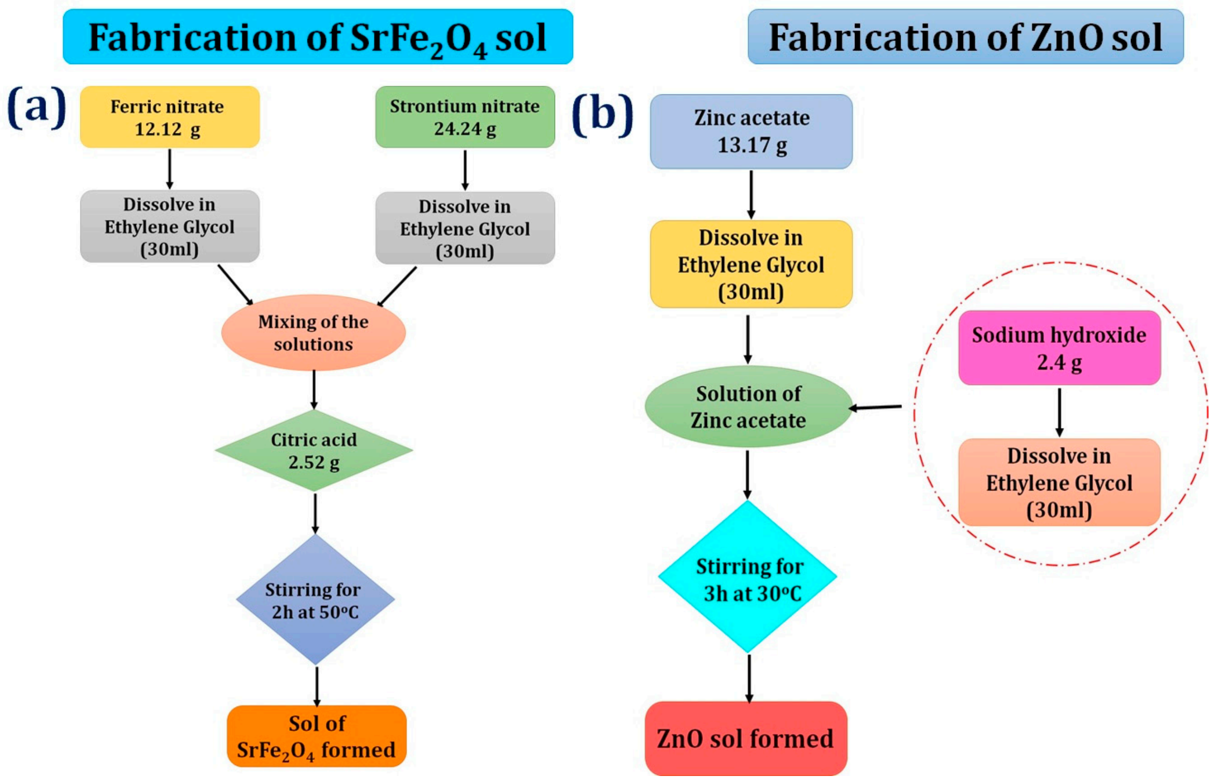

Figure S1. Schematics of various steps involved in the fabrication of sol of (**a**) $SrFe_2O_4$ and (**b**) ZnO

2.1.3 Preparation of ZnO sol

Zinc acetate dihydrate, sodium hydroxide, and ethylene glycol are used as starting materials for the preparation of ZnO sol. $Zn(CH_3CO_2)_2.2H_2O$ of 2M (13.17g) is dissolved in 30 ml of ethylene glycol under continuous stirring at 30°C till it is completely dissolved. On the other hand, the second solution of NaOH having 2M (2.4g) concentration is prepared in ethylene glycol. This solution is added drop-wise in zinc acetate solution and stirred vigorously for 3 hours at 30°C. The formation of transparent and viscous sol indicates the formation of ZnO sol and this will be further

used for the deposition of the electron transport layer (ETL). The detailed description for the fabrication of ZnO sol has been described in figure S1(b)

2.1 Morphology of the Perovskite Device:- Layer wise

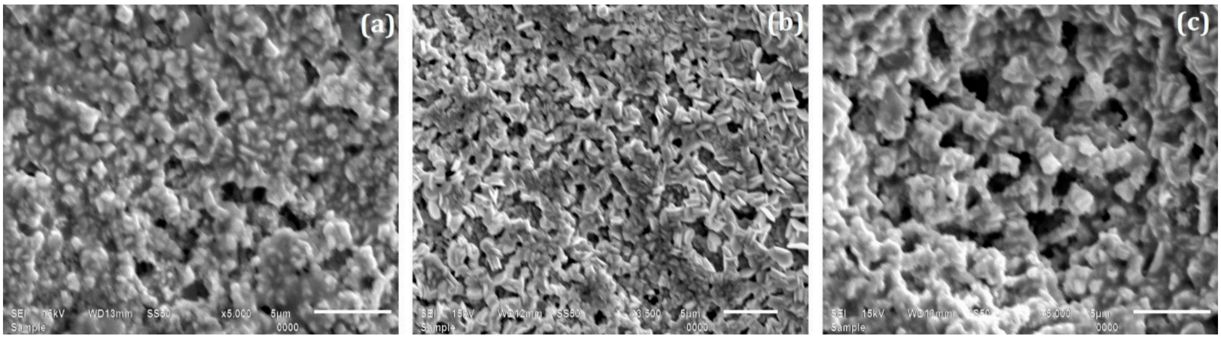

Figure S2. SEM image of the top view and surface morphology of the various layers of fabricated device (**a**) HTL $SrFe_2O_4$ (**b**) perovskite $CH_3NH_3PbI_3$ (**c**) ETL ZnO.

2.1.1 Surface roughness of layers

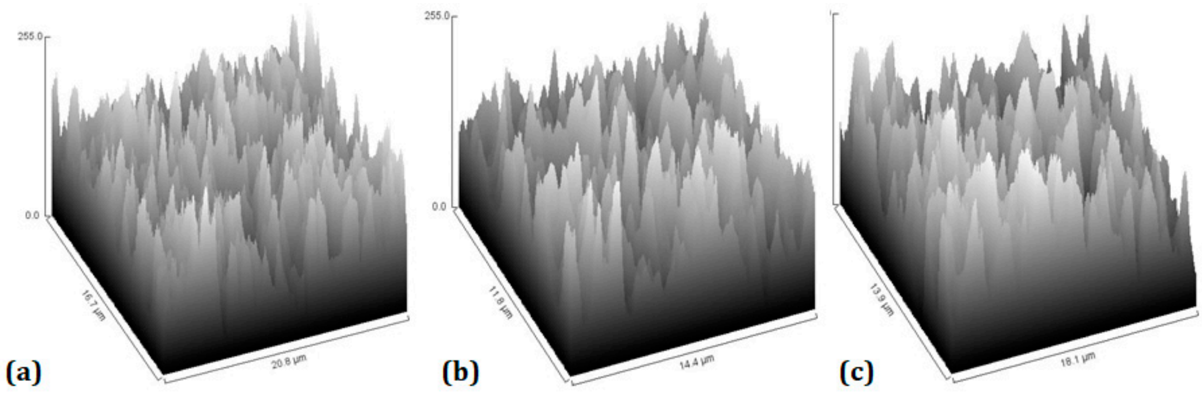

Figure S3. Surface roughness of the various layers of fabricated device (**a**) HTL $SrFe_2O_4$ (**b**) perovskite $CH_3NH_3PbI_3$ (**c**) ETL ZnO, calculated using SEM. The value of the surface roughness is around μm.

2.2 Structural and crystallographic analysis

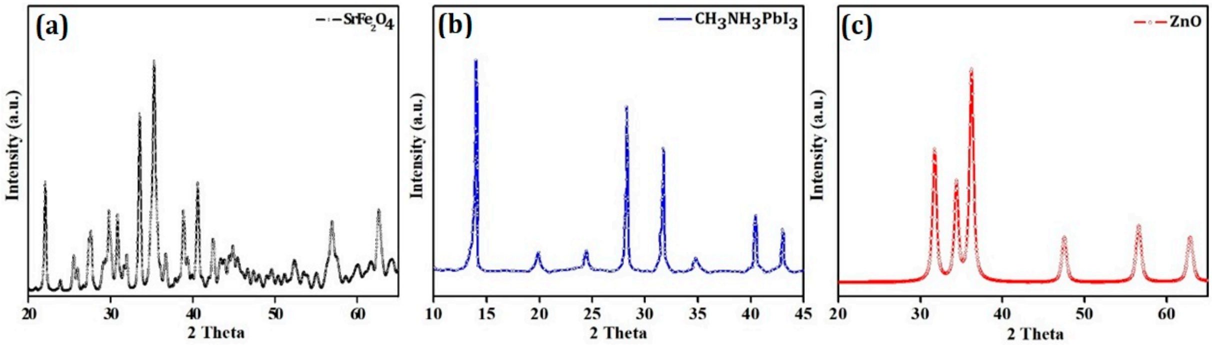

Figure S4. XRD patterns of (**a**) HTL $SrFe_2O_4$ (**b**) perovskite $CH_3NH_3PbI_3$ (**c**) ETL ZnO

Figure S4 (a), (b) and (c) show the XRD pattern of the fabricated thin films of $SrFe_2O_4$ $CH_3NH_3PbI_3$ and ZnO,respectively. It has been observed from the figure S3(a) XRD pattern of $SrFe_2O_4$ thin films are well consistent to Miller indices (120), (201), (040), (121), (220), (041), (141), (221), (240), (061), (102), (311), (042), (341), (400), (322), (371), (450), (143) while the figure S3 (c) (100), (002), (101), (102), (110), (103) indexed for ZnO, respectively. Further these XRD patterns are in well agreement with the JCPDS (Joint Committee on Powder Diffraction Standards) file number: 48-0156 for $SrFe_2O_4$ and 79-2205 for ZnO, respectively. The lattice parameters of thin films have been found to be a = 8.031, b = 18.20, c = 5.454; α =90°, β = 90 °, γ = 91.42 ° for $SrFe_2O_4$, and, a = 3.25, b = 3.25, c = 5.20; α = 90 °, β = 90 °, γ = 120° for ZnO, respectively. Figure S4 revealed the monoclinic crystal structure for $SrFe_2O_4$ with space group $P2_1/m$ while hexagonal ZnO with space group P63mc. The crystal structure of $CH_3NH_3PbI_3$ is tetragonal, figure S4 (b) with I4/mcm space group.

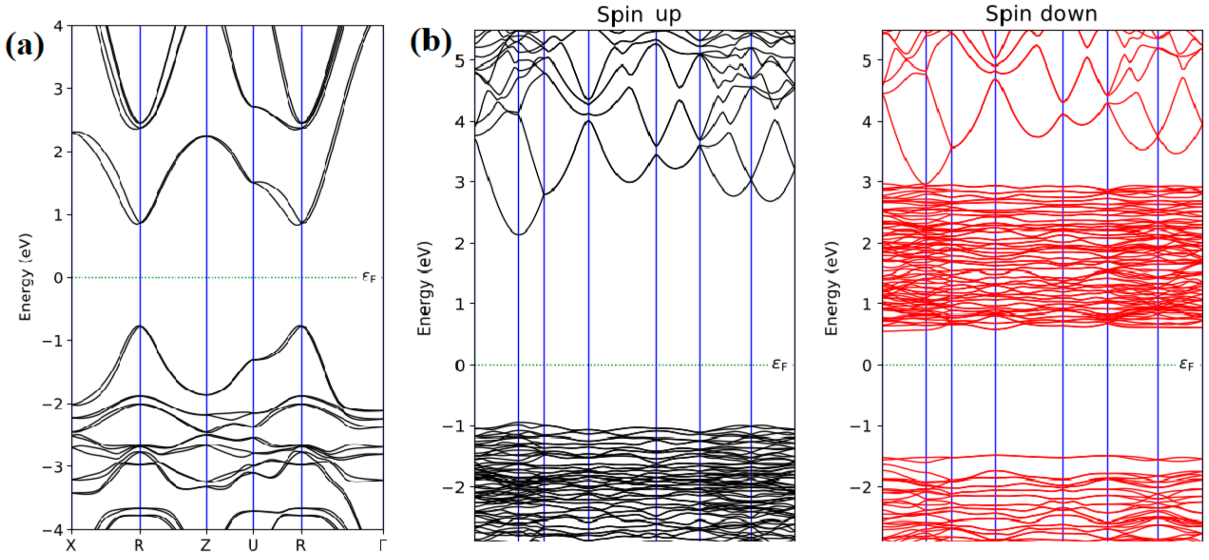

Figure S5. Electronic band structures of (**a**) perovskite $CH_3NH_3PbI_3$ and (**b**) HTL $SrFe_2O_4$

2.3 Device simulation details

The fundamental equations used during SCAPS 1-D simulation [1,2] are expressed as

$$\frac{d^2}{dx^2}\phi(x) = \frac{q}{\varepsilon_0\varepsilon_r}[p(x) - n(x) + N_D^+ - N_A^- + p_t - n_t] \quad \text{(S1)}$$

$$\frac{dJ_n}{dx} = G - R \quad \text{(S2)}$$

$$\frac{dJ_p}{dx} = G - R \quad \text{(S3)}$$

where $\phi, q, \varepsilon_0, \varepsilon_r, p, n, N_A^-, N_D^+, p_t, n_t, J_n, J_p, R \; and \; G$ denote the electrostatic potential, electric charge, vacuum, relative permittivity, the concentration of free holes, the concentration of free electrons, the dopant carrier concentration of negatively charged acceptor, the dopant carrier concentration of positively charged donor, the trapped hole concentration, trapped electron concentration, the current densities of electron and hole, the carrier recombination rate, and the generation rate. The transport of charge carrier is defined by the equations of drift and diffusion for electrons and holes with appropriate boundary conditions

$$J_n = D_n\frac{dn}{dx} + \mu_n n\frac{d\emptyset}{dx} \quad \text{(S4)}$$

$$J_p = D_p\frac{dp}{dx} + \mu_p p\frac{d\emptyset}{dx} \quad \text{(S5)}$$

where μ_n and μ_p are the mobility of electrons and mobility of the hole, respectively. According to Shockley–Read–Hall (SRH) model, the recombination rate (R) is given as [6]:

$$R_{SRH} = \sigma_{n,p} v_{th} N_t \frac{n_p - n_i^2}{n + p + 2n_i \cos\left(\frac{E_t - E_i}{KT}\right)} \quad \text{(S6)}$$

Here, $\sigma_n$and σ_p represent capture cross-sections for electrons and holes, respectively, v_{th} is thermal velocity, N_t is defect density, n and p are the concentrations of electrons and holes, n_i is the intrinsic density, E_i is the intrinsic energy level, and E_t denotes the energy level of the trap defect.

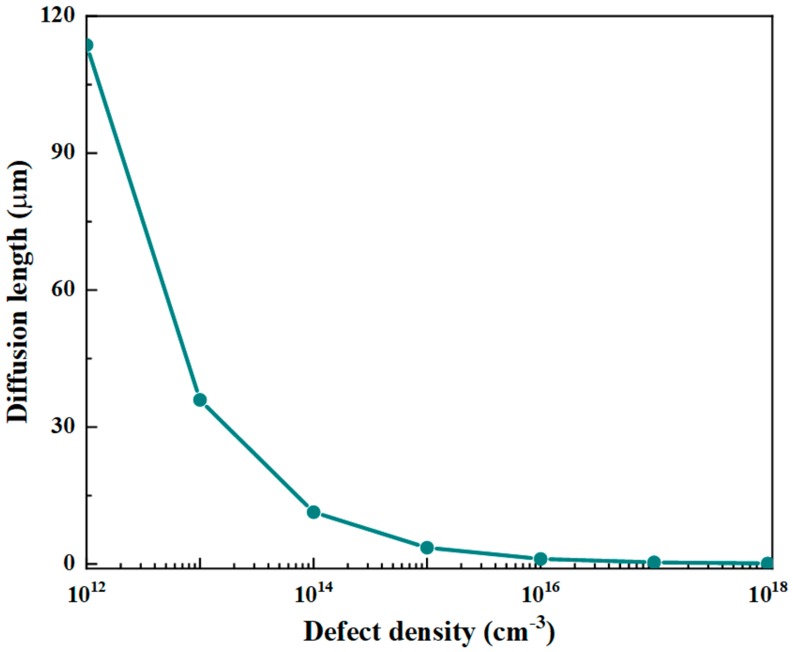

Figure S6. Carrier diffusion length versus defect density in absorber perovskite.

The diffusion length for the electron and hole can be calculated by using the following equation [6]:

$$L = \sqrt{D\tau} \quad \text{(S7)}$$

The charge carrier life time, τ, is given by

$$\tau_{n,p} = \frac{1}{\sigma_{n,p} v_{th} N_t} \quad \text{(S8)}$$

The diffusion coefficient, D, is expressed by

$$D = \frac{\mu T K_B}{q} \quad \text{(S9)}$$

K_B and μ denote the Boltzmann constant and charge carrier mobility, and q and T are the magnitude of charge and temperature in kelvin. Note that the diffusion coefficient is proportional to the mobility of charge carriers.

Table S1. Parameters for the optoelectronic simulation, E_g- Bandgap; χ_e - Electron affinity; ε_r – Permittivity; μ_n - Electron mobility; μ_p - Hole mobility; N_C - Effective density of states at conduction band (CB); N_v - Effective density of states at valence band (VB); N_D – Density of n-type doping; N_A - Density of p-type doping; *N* - Density of defects.

**Properties#**	**FTO**	**$SrFe_2O_4$**	**$CH_3NH_3PbI_3$**	**ZnO**
**E_g (eV)**	3.5	2.61	1.56	3.3
**χ_e (eV)**	4	2.5	4.4	4.2
**ε_r**	9	10	6.5	9
**μ_n (cm^2/V. s)**	20	10	50	100
**μ_p(cm^2/V. s)**	10	25	50	25
**N_C (cm^{-3})**	2.2 x10^{18}	10^{19}	10^{18}	10^{19}
**N_V (cm^{-3})**	1.8 x10^{19}	10^{19}	10^{18}	10^{19}
**N_D (cm^{-3})**	10^{19}	-	10^{9}	10^{17}
**N_A (cm^{-3})**	-	10^{16}	10^{9}	-
**N (cm^3)**	10^{14}	10^{14}	10^{15}	10^{14}

#The values of simulation parameters are adopted from theoretical and experimental works [3-5].

References

1. Rakocevic, L., Gehlhaar, R., Merckx, T., Qiu, Paetzold, U. W., Fledderus, H, Poortmans, J; Photovolt, Interconnection Optimization for Highly Efficient Perovskite Modules. IEEE J. **2017**, *7*, 404–408.
2. Stuckelberger, M., Nietzold, T., Hall, G.N., West, B, Werner, J., Niesen, B., Ballif, C. Rose, V., Fenning, D. P., Bertoni, M. I., Charge Collection in Hybrid Perovskite Solar Cells: Relation to the Nanoscale Elemental Distribution, *Ieee* J. Photovolt, **2017**, *7*, 590–597.
3. Pandey, R.; Chaujar, R, J. Technology computer aided design of 29.5% efficient perovskite/interdigitated back contact silicon heterojunction mechanically stacked tandem solar cell for energy-efficient applications. *J. Photonics Energy*. **2017**, *7(2)*: 022503.
4. Anwar F, Mahbub R, Satter SS, Ullah SM.Effect of Different HTM Layers and Electrical Parameters on ZnO Nanorod-Based Lead-Free Perovskite Solar Cell for High-Efficiency Performance. *Int. J. Photoenergy*, **2017**, 1-9.
5. A-A. Kanoun, S. Goumri-Said, M. B. Kanoun, Device design for high-efficiency monolithic two-terminal, four-terminal mechanically stacked, and four-terminal optically coupled perovskite-silicon tandem solar cells. *Int*. *J.* Energy Res., **2021**, *45*, 10538-10545.
6. W. Shockley, W. T. Read, Statistics of the Recombinations of Holes and Electrons. Phys. Rev. , **1952**, *87 (5)*: 835-842.
